# Oxidative Stress and Age-Related Tumors

**DOI:** 10.3390/antiox13091109

**Published:** 2024-09-13

**Authors:** Emma Di Carlo, Carlo Sorrentino

**Affiliations:** 1 Department of Medicine and Sciences of Aging, “G. d’Annunzio” University of Chieti-Pescara, 66100 Chieti, Italy; 2 Anatomic Pathology and Immuno-Oncology Unit, Center for Advanced Studies and Technology (CAST), “G. d’Annunzio” University of Chieti-Pescara, 66100 Chieti, Italy

**Keywords:** oxidative stress, cancer, aging, antioxidants

## Abstract

Oxidative stress is the result of the imbalance between reactive oxygen and nitrogen species (RONS), which are produced by several endogenous and exogenous processes, and antioxidant defenses consisting of exogenous and endogenous molecules that protect biological systems from free radical toxicity. Oxidative stress is a major factor in the aging process, contributing to the accumulation of cellular damage over time. Oxidative damage to cellular biomolecules, leads to DNA alterations, lipid peroxidation, protein oxidation, and mitochondrial dysfunction resulting in cellular senescence, immune system and tissue dysfunctions, and increased susceptibility to age-related pathologies, such as inflammatory disorders, cardiovascular and neurodegenerative diseases, diabetes, and cancer. Oxidative stress-driven DNA damage and mutations, or methylation and histone modification, which alter gene expression, are key determinants of tumor initiation, angiogenesis, metastasis, and therapy resistance. Accumulation of genetic and epigenetic damage, to which oxidative stress contributes, eventually leads to unrestrained cell proliferation, the inhibition of cell differentiation, and the evasion of cell death, providing favorable conditions for tumorigenesis. Colorectal, breast, lung, prostate, and skin cancers are the most frequent aging-associated malignancies, and oxidative stress is implicated in their pathogenesis and biological behavior. Our aim is to shed light on the molecular and cellular mechanisms that link oxidative stress, aging, and cancers, highlighting the impact of both RONS and antioxidants, provided by diet and exercise, on cellular senescence, immunity, and development of an antitumor response. The dual role of ROS as physiological regulators of cell signaling responsible for cell damage and diseases, as well as its use for anti-tumor therapeutic purposes, will also be discussed. Managing oxidative stress is crucial for promoting healthy aging and reducing the risk of age-related tumors.

## 1. Introduction

Oxidative stress is implicated in numerous physiological processes, including cell signaling, immune response, and the regulation of gene expression [1]. In normal conditions, the production of reactive oxygen species (ROS), such as the hydroxyl radical (^•^OH), the superoxide anion (O_2_^•−^), hydrogen peroxide (H_2_O_2_), singlet oxygen (^1^O_2_), and reactive nitrogen species (RNS, generated when nitric oxide interacts with reactive oxygen species) [2], such as nitric oxide (NO), and peroxynitrite (ONOO^−^), normally generated as by-products of the oxygen and nitrogen metabolism, is balanced by the presence of antioxidants, such as superoxide dismutase (SOD), glutathione, glutathione peroxidase (GPx), catalase, and thioredoxin, which neutralize reactive species and maintain cellular homeostasis [3]. Maintaining redox homeostasis is critical for cellular function and adaptation to environmental stressors. Moderate levels of RONS, namely oxidative eustress, are critical to maintaining, modulating, and regulating cellular functions through reversible interactions between RONS and the components of cellular signaling pathways that control those functions [4]. Excessive or uncontrolled oxidative stress, namely oxidative distress, causes cellular damage and dysfunction, contributing to the pathogenesis of age-related diseases [5]. Oxidative and nitrosative stresses are intimately linked to the aging process since the accumulation of damage to macromolecules such as DNA, RNA, proteins, and lipids contributes to cellular senescence and increased susceptibility to age-related pathologies, including neurodegenerative and cardiovascular diseases, diabetes, inflammatory conditions, and cancer [6,7]. The progressive increase in blood levels of factors secreted by senescent cells, known as the senescence-associated secretory phenotype (SASP), promotes chronic inflammation and low-grade chronic damage, which accelerate the senescence of immune cells, resulting in a weakened immune function and an inability to clear senescent cells and inflammatory factors, which creates a vicious cycle of inflammation and senescence, namely “inflammaging” [8]. The mechanisms underlying the two-way links of oxidative distress with inflammation and aging, which impact tumorigenesis and the response to anticancer therapies, will be explored below.

## 2. Antioxidant and Oxidative Stress Driving Genes

Several genes are implicated in promoting oxidative stress, either by enhancing the production of ROS or by impairing antioxidant defenses [3,9]. Antioxidant coding genes are crucial in protecting cells from damage caused by oxidative stress, which is the result of an imbalance between ROS and the body’s ability to detoxify them [10]. These genes encode proteins that function as antioxidants, repair enzymes, and regulators of redox signaling pathways.

*Key genes involved in oxidative stress and their functions are outlined below.*

**NADPH oxidases (NOX)**. NADPH oxidases are a family of enzymes, including NOX1, NOX2, NOX3, NOX4, and NOX5, that generate ROS as part of the innate immune response and various signaling pathways [11,12]. NOX enzymes transfer electrons from NADPH to molecular oxygen, producing superoxide radicals (O_2_^•−^) and other ROS. The dysregulation of NOX activity is implicated in oxidative stress-related diseases.

**Nitric Oxide Synthases (NOS).** Enzymes such as NOS1, NOS2 (iNOS), and NOS3 (eNOS) produce NO, which has antioxidant properties and can scavenge ROS. Under pathological conditions, such as in the presence of high levels of superoxide, NO can react with superoxide to form ONOO^−^, a highly reactive and damaging species [13]. This reaction reduces the availability of NO, leading to endothelial dysfunction and contributing to oxidative stress [14]. Factors leading to this uncoupling include a deficiency in essential cofactors like tetrahydrobiopterin (BH4) or substrates like L-arginine. Uncoupled NOS contributes to a vicious cycle of increased ROS production and further oxidative stress.

**Arachidonate Lipoxygenases (ALOX).** The ALOX family includes several isoforms, such as 5-LOX, 12-LOX, and 15-LOX. Each isoform catalyzes the oxidation of specific positions on polyunsaturated fatty acids to produce lipid peroxides, leading to different biological effects. During the metabolism of fatty acids, ALOX enzymes, such as ALOX5, generate lipid hydroperoxides, which contribute to oxidative stress, inflammation, and cancer [15].

**Cytochrome P450 Enzymes (CYP).** The major families involved in drug metabolism are CYP1, CYP2, and CYP3. CYP enzymes are primarily located in the liver and are involved in the metabolism of xenobiotics and endogenous compounds such as steroid hormones, fatty acids, and bile acids. Some CYP enzymes, such as CYP2E1, generate ROS as by-products of their catalytic activity, contributing to oxidative stress [16,17].

**Xanthine Dehydrogenase (XDH)/Xanthine Oxidoreductase (XOR)**. XDH and XOR are enzymes that play critical roles in purine metabolism. They catalyze the oxidation of hypoxanthine into xanthine and subsequently of xanthine into uric acid. Under certain conditions, such as oxidative stress or proteolytic cleavage, XDH can be converted into xanthine oxidase (XO), which produces superoxide and hydrogen peroxide during purine degradation, contributing to oxidative stress [18].

**Hypoxia-Inducible Factor 1 Alpha (HIF1A).** HIF1A is a key transcription factor that responds to changes in cellular oxygen levels. Under normoxic conditions, HIF1A is hydroxylated by prolyl hydroxylase domain enzymes (PHDs), marking it for ubiquitination and proteasomal degradation [19]. Under hypoxic conditions, the hydroxylation is inhibited, stabilizing HIF1A and allowing it to translocate into the nucleus where it dimerizes with HIF1B and activates target genes involved in glycolysis and angiogenesis. It can also promote ROS production, contributing to oxidative stress [20].

These genes are deeply involved in the molecular mechanisms driving oxidative stress, which is implicated in age-related diseases, including chronic inflammatory conditions and cancer.

*Key genes driving antioxidant activity, and their functions are outlined below.*

**SOD**. SOD enzymes catalyze the dismutation of O_2_^•−^ into oxygen (O_2_) and hydrogen peroxide (H_2_O_2_), thereby protecting cells from superoxide-mediated oxidative damage. There are several isoforms of SOD, including cytosolic SOD1 (Cu/Zn-SOD), mitochondrial SOD2 (Mn-SOD), and extracellular SOD3 (Ec-SOD). Mutations in SOD1 are associated with familial amyotrophic lateral sclerosis (ALS) [21,22]. Mutant SOD1 proteins can misfold and aggregate, leading to mitochondrial dysfunction and increased ROS production [23].

**Catalase (CAT)**. Catalase is an enzyme that catalyzes the decomposition of hydrogen peroxide (H_2_O_2_) into water (H_2_O) and O_2_, thereby neutralizing H_2_O_2_ and preventing its harmful effects. Catalase is primarily localized within peroxisomes and plays a critical role in cellular antioxidant defense mechanisms [24].

**GPX**. Glutathione peroxidases are a family of enzymes that catalyze the reduction of hydroperoxides, including hydrogen peroxide (H_2_O_2_), using reduced glutathione (GSH) as a cofactor. GPX enzymes protect cells from oxidative damage by scavenging peroxides and maintaining cellular redox balance [25].

**Glutathione reductase (GSR)**: Glutathione reductase is an enzyme that catalyzes the reduction of oxidized glutathione (GSSG) to its reduced form (GSH), which serves as a major cellular antioxidant. GSR plays a crucial role in recycling GSH and maintaining cellular redox homeostasis [26].

**Nuclear factor erythroid 2-related factor 2 (Nrf2)**. Nrf2 is a transcription factor that regulates the expression of antioxidant and detoxification genes in response to oxidative stress [27]. Under basal conditions, Nrf2 is sequestered in the cytoplasm by Keap1 (Kelch-like ECH-associated protein 1). Upon exposure to oxidative stress, Nrf2 is released from Keap1, translocates to the nucleus, and activates the transcription of antioxidant response element (ARE)-containing genes, such as those encoding antioxidant enzymes, phase II detoxification enzymes, and NADPH-generating enzymes [28].

**Heme oxygenase-1 (HO-1).** Heme oxygenase-1 is an enzyme that catalyzes the degradation of heme into biliverdin, carbon monoxide (CO), and iron. HO-1 induction is a key adaptive response to oxidative stress and inflammation, serving as a cytoprotective mechanism against oxidative damage and cellular stress [29].

**Peroxiredoxins (PRX)**. Peroxiredoxins are a family of antioxidant enzymes that catalyze the reduction of peroxides, including hydrogen peroxide (H_2_O_2_), alkyl hydroperoxides, and peroxynitrite. PRX enzymes utilize thioredoxin or glutathione as reducing equivalents to detoxify peroxides and maintain cellular redox balance [30].

These genes represent a subset of the many components involved in cellular antioxidant defense mechanisms and oxidative stress responses. Their coordinated regulation ensures that cells can effectively neutralize reactive oxygen species and mitigate oxidative damage to maintain cellular homeostasis.

## 3. Role of Oxidative Stress in Normal Cell Signal Transduction and Homeostasis

Reactive oxygen species play a dual role in biological systems. While they are often associated with harmful effects leading to cell damage and disease, at physiological levels, ROS play crucial roles as second messengers in many intracellular signaling cascades aimed at maintaining cells in homeostasis with their microenvironment [31]. 

At low to moderate levels, **ROS act as secondary messengers in various signaling pathways**. They modulate the activity of key proteins by oxidizing specific thiol groups on cysteine residues, leading to reversible modifications that can alter protein function. 

In the context of the Epidermal Growth Factor Receptor (EGFR) pathway, ROS are generated upon the binding of growth factors like EGF to their receptors. The activation of NOX leads to the production of superoxide, which is converted into H_2_O_2_. In turn, H_2_O_2_ modulates the activity of protein tyrosine phosphatases (PTPs) by oxidizing their cysteine residues, leading to the persistence of phosphorylation events and thus amplifying the growth factor signaling. This regulation can affect cellular processes such as proliferation, migration, and differentiation [32,33].

In the context of the Hypoxia-Inducible Factor (HIF) pathway, ROS are produced in the mitochondria in response to hypoxia. These ROS stabilize HIF-1α by inhibiting prolyl hydroxylases, which usually mark HIF-1α for degradation under normal oxygen conditions. Stabilized HIF-1α translocates to the nucleus, where it activates the transcription of genes involved in angiogenesis (e.g., vascular endothelial growth factor, VEGF), erythropoiesis, and glycolysis, helping cells adapt to hypoxic conditions [34].

**ROS can modulate the activity of kinases**, such as protein tyrosine kinases and mitogen-activated protein kinases (MAPKs), which are involved in controlling cell proliferation, differentiation, and apoptosis [35]. ROS-mediated activation of kinases in the MAPK pathway, such as extracellular signal-regulated kinases (ERKs), c-Jun N-terminal kinases (JNKs), and p38 MAPKs, occurs through the oxidative modification of cysteine residues in upstream kinases and phosphatases, leading to cellular responses like proliferation, differentiation, stress responses, and apoptosis [35].

**ROS regulate the activity of transcription factors** [36], including, ***a. Nuclear Factor-kappa B (NF-κB).*** NF-κB, a transcription factor involved in inflammatory and immune responses, can be activated by ROS. The oxidative modification of inhibitory proteins like IκB leads to the release and activation of NF-κB, allowing it to translocate to the nucleus and initiate gene expression. ***b. Activator Protein-1 (AP-1).*** ROS can enhance the activity of AP-1, which regulates gene expression related to proliferation and differentiation. ***c. Nrf2.*** Under normal conditions, Nrf2 is bound to its inhibitor Keap1 and targeted for degradation. Under oxidative stress, Nrf2 dissociates from Keap1, translocates to the nucleus, and activates the expression of genes involved in antioxidant defense and detoxification, thus restoring homeostasis. 

Cells maintain redox homeostasis by balancing ROS production and scavenging. This balance is critical for cellular adaptation to environmental changes. ROS can induce adaptive responses, such as the upregulation of antioxidant defense systems (e.g., glutathione peroxidase, superoxide dismutase), which help restore redox balance.

**Controlled ROS levels are involved in the regulation of cell cycle progression, proliferation, and differentiation.** For example, H_2_O_2_ has been shown to regulate the transition between different phases of the cell cycle, influencing cell division. ROS influence the expression and activity of key cell cycle regulators, such as cyclins, cyclin-dependent kinases (CDKs), and CDK inhibitors. For instance, moderate ROS levels can upregulate cyclin D1, driving the transition from the G1 to the S phase of the cell cycle, thereby promoting DNA synthesis and cell division. ROS can activate or inhibit Notch signaling depending on the context, influencing differentiation outcomes [37,38].

**ROS can influence calcium signaling by modulating calcium channels and transporters.** Calcium is a vital second messenger in numerous cellular processes, and ROS can influence intracellular calcium levels by modulating calcium channels and pumps, such as the ryanodine receptor (RyR) and the inositol 1,4,5-trisphosphate receptor (IP3R). ROS-induced calcium release from the endoplasmic reticulum can activate various calcium-dependent signaling pathways, influencing processes like muscle contraction, neurotransmitter release, and cell death [39,40].

**ROS play a pivotal role in apoptosis** by influencing signaling pathways that regulate cell survival and death. For example, ROS can activate pro-apoptotic proteins like Bax and Bak, leading to mitochondrial outer membrane permeabilization (MOMP) and the release of cytochrome c, which activates caspases to execute cell death. ROS also modulate signaling through the p53 pathway, which can lead to cell cycle arrest or apoptosis depending on the context [41].

**ROS are critical regulators of autophagy**, a cellular degradation process that helps maintain homeostasis by removing damaged organelles, misfolded proteins, and pathogens. Autophagy can be induced by various stressors, including nutrient deprivation, hypoxia, and oxidative stress. By influencing key signaling pathways and transcription factors, ROS can induce and regulate the autophagic process, promoting cell survival under stress conditions by clearing damaged components and maintaining cellular homeostasis [42].

**ROS are involved in signaling processes that regulate immune cell functions**, such as phagocytosis [43] and the production of cytokines [44]. Low levels of ROS contribute to the regulation of immune responses, ensuring that immune cells respond appropriately to pathogens without causing excessive tissue damage. For instance, upon activation of TLR4 by lipopolysaccharide (LPS), ROS are produced by NOX and mitochondria. These ROS participate in the activation of downstream signaling pathways, including the NF-κB and MAPK pathways, which lead to the production of pro-inflammatory cytokines and other immune responses [45,46].

In summary, oxidative stress, when tightly regulated, plays an essential role in normal cell signal transduction and homeostasis. It acts as a critical regulator of various cellular processes, including redox signaling, proliferation, differentiation, and immune responses. The fine balance between ROS production and antioxidant defense systems ensures that cells can respond to environmental cues and maintain homeostasis without tipping into pathological states associated with excessive oxidative damage.

## 4. Oxidative Stress and Ageing

Oxidative stress promotes aging through a series of interconnected biological processes, and age is one of the main risk factors for cancer [47]. The following subsections present detailed explanations of the reasons behind this connection.

**Cellular and DNA Damage and Mutations.** Over the years, oxidative stress and DNA damage caused by life-long exposure to endogenous metabolic insults (e.g., free radicals) and exogenous factors (e.g., UV irradiation, foods, etc.) accumulate. ROS can react with DNA, leading to various types of damage, including base modifications, single-strand breaks, double-strand breaks, and mutagenic lesions such as the formation of 8-oxo-2′-deoxyguanosine (8-oxo-dG), which can lead to erroneous base pairing during DNA replication. 8-oxo-dG-induced DNA damage can activate cellular senescence [48], a state of permanent growth arrest, resulting in the secretion of inflammatory factors, which further contribute to tissue degeneration and aging. The accumulation of 8-oxo-dG in DNA leads to increased genomic instability, which is a hallmark of aging and age-related diseases. Overall, the accumulation of DNA damage can result in faulty protein production and disrupted cellular processes, which impair cellular functions and contribute to genomic instability, promoting aging processes and increasing the risk of developing cancer [49].

**Mitochondrial Dysfunction.** Mitochondria are the powerhouses of the cell and are a major source of ROS. Excessive ROS production can damage mitochondrial components, including lipids, proteins, and mitochondrial DNA (mtDNA), leading to mitochondrial dysfunction. Mitochondrial membranes are particularly vulnerable to lipid peroxidation, which can impair the function of membrane-bound enzymes and ion channels. Damage to mtDNA can lead to mutations. Unlike nuclear DNA, mtDNA is more susceptible to oxidative damage due to its proximity to the electron transport chain and the lack of protective histones [50]. Accumulated mutations in mtDNA can disrupt the synthesis of key proteins required for the electron transport chain (ETC), further impairing mitochondrial function and increasing ROS production in a vicious cycle that accelerates cellular aging and reduces energy production [51].

**Telomere Shortening.** Telomeres are protective caps at the ends of chromosomes that shorten with each cell division. Oxidative stress accelerates telomere shortening through different mechanisms [52,53]: **1. Direct Oxidative Damage to Telomeres.** Telomeres are particularly susceptible to oxidative damage due to their high guanine content (G-rich sequences). Guanine is the most easily oxidized of the four DNA bases, and oxidation leads to the formation of 8-oxoguanine (8-oxoG), a mutagenic lesion. ROS-induced damage at telomeres results in single-strand breaks and base modifications. DNA repair at telomeres is less efficient compared to other genomic regions, due to the limited access of DNA repair machinery at these specialized chromosomal ends, leading to unresolved damage and faster telomere attrition [54]. **2. Impaired Telomerase Function.** Oxidative stress can impair the function of telomerase (the enzyme responsible for adding telomeric repeats to the ends of chromosomes) through several pathways: ***a. telomerase inhibition***. ROS can directly damage the telomerase RNA component (TERC) or the reverse transcriptase (TERT) catalytic subunit, leading to reduced telomerase activity and accelerated telomere shortening; ***b. decreased telomerase recruitment***. Oxidative stress can interfere with the recruitment of telomerase to telomeres, further preventing telomere elongation. **3. Telomere Loop (T-loop) Disruption.** Telomeres form a protective structure called the T-loop, which hides the chromosome end from being recognized as a DNA break [55]. Oxidative stress can disrupt this structure, exposing the telomere ends and making them more prone to degradation and fusion. **4. Replication Stress at Telomeres.** ROS can induce replication stress, leading to stalled replication forks, particularly at telomeres. Telomeres are difficult to replicate because of their repetitive sequence and secondary structures like G-quadruplexes, which make them more vulnerable to replication fork stalling and collapse [56]. Fork stalling can result in incomplete replication of telomeres, leading to shortened telomeres with each cell division.

When telomeres become critically short, cells enter a state of senescence or apoptosis, contributing to tissue aging and dysfunction, or leading to genomic instability by causing chromosome end-to-end fusions, breakage-fusion-bridge cycles, and aneuploidy. This genomic instability can drive the progression of cancer [57,58].

**Protein Oxidation and Aggregation.** ROS can affect protein structure and function. ROS can directly modify the side chains of amino acids, leading to the formation of disulfide bonds (in the case of cysteine) or other oxidized derivatives, which can disrupt the protein’s tertiary and quaternary structure. ROS can attack the carbon backbone of amino acids, leading to the introduction of carbonyl groups (C=O) into the protein structure, resulting in a loss of protein function or increased susceptibility to proteolytic degradation. ROS can either disrupt or form incorrect disulfide linkages, which can lead to the misfolding or aggregation of proteins. ROS can break peptide bonds within the protein, leading to fragmentation and loss of structural integrity [59]. Oxidized proteins can lose their enzymatic activity or alter cellular signaling when involved in signaling pathways. The impairment of structural integrity makes proteins susceptible to aggregation. Protein aggregation is sufficient to activate the stress response and inflammation, impairing protein synthesis and quality control mechanisms, which are associated with age-related diseases [60] and are implicated in anti-tumor therapy resistance [61].

**Inflammation.** Oxidative stress can trigger inflammatory responses, through the activation of redox-sensitive transcription factors such as NF-κB and AP-1, leading to chronic inflammation. “Inflammaging” refers to the chronic, low-grade inflammation that typically accompanies aging, which is characterized by increased levels of pro-inflammatory markers in the blood and tissues [8] which feeds oxidative stress, creating a feedback loop that exacerbates cellular damage. This phenomenon is thought to be driven by cellular senescence, mitochondrial dysfunction, altered immune response, and epigenetic changes, which promote cancer development and the response to anti-cancer immunotherapy [62,63].

**Cellular Senescence.** Cells that experience significant oxidative damage may enter a state of senescence, where they no longer divide but remain metabolically active. Senescent cells secrete pro-inflammatory cytokines, growth factors (including IL-6, IL-8, TNF-α, MCP-2/CCL8, MCP-1/CCL2, and GRO-α/CXCL1), proteases (such as matrix metalloproteinases, MMPs), and other signaling molecules known as the senescence-associated secretory phenotype (SASP). SASP factors can damage surrounding tissues and disrupt normal cellular function, contributing to aging and inflammation [64] and creating a tumorigenic microenvironment.

**Impaired Autophagy.** Autophagy is a cellular process that degrades and recycles damaged organelles, misfolded proteins, and other cellular debris. This process is crucial for maintaining cellular homeostasis, especially under stress conditions. Oxidative stress can impair autophagy, leading to the accumulation of damaged cellular components. This impairment further exacerbates cellular dysfunction and contributes to the aging process [65]. The decline in autophagy with age contributes to the accumulation of cellular damage, increasing the risk of cancer development.

**Antioxidant Defense Decline.** Aging is associated with a decline in antioxidant defense mechanisms, including enzymatic antioxidants such as SOD, catalase, and glutathione peroxidase, as well as non-enzymatic antioxidants like vitamins C and E, glutathione, and flavonoids. Reduced antioxidant capacity contributes to increased susceptibility to oxidative damage and impaired cellular homeostasis with age [66,67].

In summary, oxidative stress promotes aging by causing cumulative damage to DNA, proteins, lipids, and cellular organelles. This damage leads to mitochondrial dysfunction, telomere shortening, protein aggregation, chronic inflammation, cellular senescence, and impaired autophagy. Together, these processes drive the decline in cellular and tissue function that characterizes aging.

## 5. Oxidative Stress and Tumorigenesis

Oxidative stress is implicated in the development and progression of age-related cancers by promoting DNA damage, inflammation, cellular senescence, mitochondrial dysfunction, and impaired antioxidant defenses [68,69]. 

The link between oxidative stress and DNA damage, which plays a significant role in aging and cancer, arises from the imbalance between the production of ROS and the body’s ability to neutralize them with antioxidants. When the levels of highly reactive molecules, such as free radicals like O_2_^•−^, ^•^OH, and non-radical molecules like H_2_O_2_, exceed the capacity of antioxidant defenses, they can attack cellular components, including DNA, and cause multiple **types of damage**: **a. Single-strand breaks (SSBs).** ROS can cause breaks in one strand of the DNA helix, leading to replication errors or mutations. Mutations leading to oncogene activation and tumor suppressor gene [70] inactivation can drive tumorigenesis [71]. **b. Double-strand breaks (DSBs).** More severe than SSBs, DSBs occur when both strands of the DNA helix are broken, which can lead to chromosomal rearrangements or cell death if not repaired. DNA damage sensors, such as the Ataxia-Telangiectasia Mutated (ATM) kinase, which is a critical sensor for double-strand breaks, and Poly ADP-Ribose Polymerase (PARP), which recognizes single-strand breaks, are the first responders in the DNA repair process, and their functions are critical to the maintenance of genome integrity. **c. Base modifications.** ROS can oxidize DNA bases, such as the conversion of guanine to 8-oxo-7,8-dihydroguanine (8-oxoG), one of the most common markers of oxidative DNA damage. This can lead to mispairing during replication and mutagenesis [72]. **d. DNA cross-linking.** ROS can also induce covalent bonding between DNA strands or between DNA and proteins, impairing normal cellular processes [73]. 

To address oxidative DNA damage, cells have evolved several **DNA repair mechanisms** [74] namely, **a. Base excision repair (BER).** This pathway is the primary repair mechanism for oxidative base modifications like 8-oxoG. Enzymes such as glycosylases recognize and remove damaged bases, and DNA polymerase fills the gap. **b. Nucleotide excision repair (NER).** This pathway repairs bulky DNA lesions, such as those caused by cross-linking. **c. Non-homologous end joining (NHEJ) and homologous recombination (HR).** These pathways repair DSBs, with HR being the more accurate of the two as it uses a sister chromatid as a template, ensuring error-free repair [75]. However, if not properly repaired, DNA damage can lead to several adverse outcomes, including apoptosis or necrosis, genomic instability, and mutations. 

**Figure 1:**
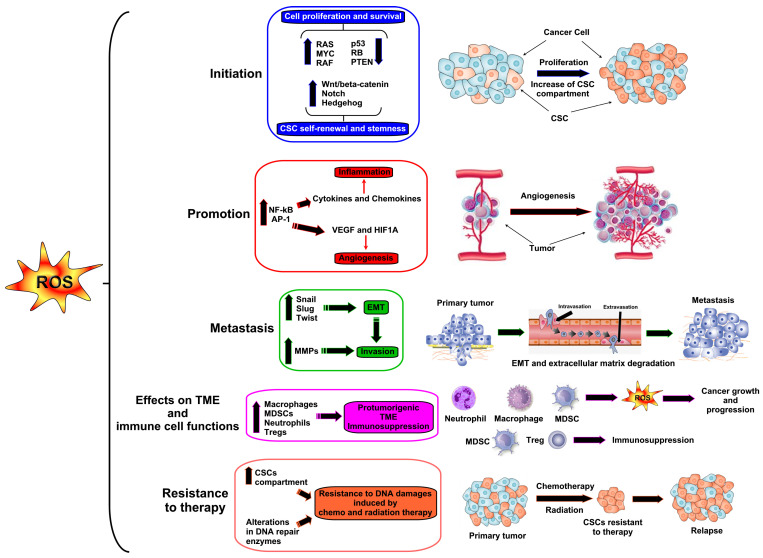
Role of oxidative stress in cancer development and progression. Reactive oxygen species (ROS) may contribute to tumor initiation, progression, metastasis, and therapy resistance by activating signaling pathways involved in cancer cell proliferation and metastatization, angiogenesis, and expansion of the cancer stem cell (CSC) compartment. Furthermore, oxidative stress plays a key role in the regulation of immune responses in the tumor microenvironment (TME). Original image created using Microsoft PhotoDraw (version 2.0.0.0822). MDSCs: myeloid-derived suppressor cells. Tregs: T regulatory cells.

Accumulation of mutations in genes involved in cell cycle regulation, DNA repair, and tumor suppression increases the risk of age-related cancers, such as breast, colorectal, prostate, and lung cancers and hematological malignancies such as non-Hodgkin lymphoma, multiple myeloma, and leukemia [76]. Oxidative stress plays a multifaceted role in all stages of tumorigenesis [76] by promoting DNA damage and mutations, which foster cell proliferation, angiogenesis, invasion, metastasis, and resistance to therapy [77,78], as outlined below and in Figure 1. 

**Initiation**. ROS can cause DNA strand breaks, base modifications, and chromosomal rearrangements, leading to mutations in oncogenes, tumor suppressor genes, and genes involved in DNA repair, which promote uncontrolled cell proliferation and the development of pre-cancerous lesions [77]. Oncogenes such as RAS, MYC, and RAF can be activated by oxidative stress through mutations or epigenetic modifications, leading to cell proliferation and survival. By contrast, tumor suppressor genes such as TP53 (p53), RB, and PTEN can be inactivated, either through direct mutations or through signaling pathways that degrade these proteins [79]. Oxidative stress also supports the cancer stem cell (CSC) compartment, which contributes to tumor initiation, progression, metastasis, and therapy resistance by activating signaling pathways involved in self-renewal and pluripotency. For example, the ROS-mediated activation of signaling pathways, such as Wnt/β-catenin, Notch, and Hedgehog, can enhance CSC self-renewal capacity and maintain their stemness properties [80]. The accumulation of mutations in these genes increases the risk of age-related cancers [67].

**Promotion**. Chronic exposure to oxidative stress promotes tumor growth and progression by inducing genomic instability, enhancing cell proliferation, and fostering a pro-tumorigenic microenvironment [81]. ROS-mediated activation of signaling pathways, such as NF-κB and AP-1, stimulates the expression of genes involved in inflammation, angiogenesis, and cell survival. ROS produced during oxidative stress can activate inflammatory signaling pathways, leading to the production of cytokines and chemokines that promote inflammatory cell recruitment. Intratumorally recruited myeloid-derived suppressor cells (MDSCs), macrophages, and neutrophils can produce ROS, further exacerbating oxidative stress and creating a pro-tumorigenic milieu that supports cancer growth and progression [82]. Oxidative stress-dependent inactivation of tumor suppressor genes and the activation of oncogenes contribute to tumor progression [79].

**Angiogenesis**. Oxidative stress promotes angiogenesis, the formation of new blood vessels, which is essential for supplying nutrients and oxygen to growing tumors [83,84]. ROS stimulate the expression of angiogenic factors, such as VEGF and HIF1A, promoting endothelial cell proliferation and vessel sprouting [85]. Increased angiogenesis facilitates tumor growth, invasion, and metastasis, contributing to the progression of cancer [86].

**Metastasis**. Oxidative stress plays a role in the metastatic spread of cancer cells to distant sites. ROS-mediated activation of epithelial-to-mesenchymal (EMT)-associated transcription factors, such as Snail, Slug, and Twist, contributes to the invasive phenotype of CSCs and facilitates metastatic dissemination [87]. Additionally, oxidative stress promotes the activation of proteases involved in extracellular matrix degradation, facilitating cancer cell invasion and metastasis [88].

**Effects on the tumor microenvironment (TME) and immune cell functions**. Immuno-senescence contributes to a higher incidence of cancer due to reduced immune surveillance and impaired antitumor immunity [89], to which oxidative stress contributes [90,91]. ROS can influence the activation and differentiation of immune cells. Moderate levels of ROS are necessary for T cell activation, but excessive ROS can cause T cell exhaustion and apoptosis. High levels of ROS in the TME can lead to immunosuppression by promoting regulatory T cells and MDSCs. 

**Resistance to therapy**. High levels of ROS are mostly involved in programmed cell death and/or apoptosis [92]. Radiation therapy kills cancer cells by generating high levels of ROS, which contributes to inducing severe DNA damages and cancer cell death. However, oxidative stress can contribute to the development of resistance to chemotherapy and radiation therapy in cancer cells since the self-renewal capability of CSCs, the main responsible for tumor growth and progression, is firmly regulated by ROS levels [93,94]. In quiescent stem cells, very low levels of ROS may be indispensable for their stemness maintenance; however, physiological ROS levels may encourage stem cell proliferation and/or differentiation [95]. CSCs also exhibit increased resistance to oxidative stress-induced cell death compared to non-CSCs. This resistance is attributed to elevated expression of antioxidant enzymes such as SOD, catalase, and glutathione peroxidase, which help CSCs neutralize ROS and protect them from oxidative damage. As a result, CSCs can survive under conditions of oxidative stress induced by chemotherapy, radiation therapy, or the TME [92,96]. Moreover, oxidative stress-induced alterations in DNA repair pathways can reduce the efficacy of DNA-damaging agents, further contributing to therapy resistance.

**Interplay with immunotherapy**. Chemotherapeutics, such as oxaliplatin, bleomycin, and doxorubicin, can induce ROS-mediated immunotherapy. Excessive ROS promote the immunogenic cell death (ICD) of tumors, providing a potential antigenic stimulation for the immune system [97]. Studies suggest that ROS can increase the infiltration of dendritic cells (DCs) and T cells in the TME and transform “cold” (low immunogenicity) tumors into “hot” (high immunogenicity) tumors, thereby increasing a more effective antitumor immune response [98,99]. Nevertheless, it has been reported that, in breast cancer, the elimination of ROS alleviates the immunosuppressive ICD in the TME and increases antitumor immunity and T-lymphocyte infiltration, resulting in a potent antitumor effect [100]. Collectively, these contradictory results suggest that ICD can be induced by modulating ROS to obtain enhanced immunotherapy.

Understanding the mechanisms underlying the interplay of oxidative stress with both the immune environment and CSCs is essential for developing effective therapeutic approaches to target immune-resistant and aggressive tumor cell populations. Strategies aimed at reducing oxidative stress and its effects may help mitigate cancer risk and improve outcomes in aging populations.

## 6. Antioxidants in the Prevention and Treatment of Age-Related Cancer

Antioxidant compounds have garnered considerable interest in cancer prevention and therapy due to their ability to neutralize free radicals, reduce oxidative stress, and mitigate some of the cellular damage associated with aging processes and the risk of developing cancer [101]. Here, we provide an overview of the current understanding of the role of antioxidant compounds in cancer prevention and therapy resulting from ongoing research and completed studies.

### 6.1. Antioxidant Mechanisms of Action

Antioxidants scavenge free radicals, preventing oxidative damage to proteins, lipids, and DNA that can lead to mutations and cancer development. Antioxidants modulate signaling pathways involved in cell proliferation, apoptosis, and inflammation, all of which are relevant to cancer development and progression.

Recent clinical trials have found that antioxidant supplementation can significantly improve certain immune responses [102]. Vitamins C and E, selenium, and zinc play vital roles in supporting the proper functioning of the immune system [103]. Specifically, supplementation with vitamins C, E, and A or beta-carotene increased the activation of cells involved in tumor immunity in the elderly [104,105].

### 6.2. Types of Antioxidants

While endogenous antioxidants are produced by the body and include enzymes like SOD, catalase, and glutathione peroxidase, exogenous antioxidants are obtained from the diet and supplements, as follows.

*Vitamins.* Vitamin C is found in citrus fruits, strawberries, bell peppers, and broccoli. It helps regenerate other antioxidants and is crucial for skin health and immune function. Vitamin E is found in nuts, seeds, spinach, and vegetable oils. It protects cell membranes from oxidative damage.

*Carotenoids.* Beta-carotene is found in carrots, sweet potatoes, and spinach. It is a precursor to vitamin A and plays a role in vision and immune function. Lycopene is found in tomatoes, watermelon, and pink grapefruit. It has been linked to a reduced risk of certain cancers.

*Polyphenols.* Flavonoids are found in berries, tea, onions, and apples. They have anti-inflammatory and anti-carcinogenic properties. Resveratrol is found in red wine, grapes, and peanuts. It is known for its potential cardiovascular benefits.

*Minerals.* Selenium is found in Brazil nuts, seafood, and meats. It is a component of antioxidant enzymes like glutathione peroxidase. Zinc is found in meat, shellfish, legumes, and seeds. It helps maintain the function of antioxidant enzymes.

### 6.3. Evidence from Research and Clinical Studies

*Epidemiological Studies.* Many studies suggest a correlation between high dietary intake of antioxidants and a reduced risk of certain cancers. For example, a diet rich in fruits and vegetables is associated with a lower risk of cancers, such as colorectal, prostate, and lung cancer [106,107].

*Clinical Trials.* The results of clinical trials are mixed. Some studies have shown benefits, while others have not demonstrated significant effects or have even suggested potential harm in certain populations. For instance, the **ATBC (Alpha-Tocopherol, Beta-Carotene Cancer Prevention) Study** found that beta-carotene supplementation increased the incidence of lung cancer in smokers. Conversely, the **SELECT (Selenium and Vitamin E Cancer Prevention Trial)** did not show a protective effect of selenium and vitamin E supplements against prostate cancer and indicated an increased risk of prostate cancer with vitamin E supplementation [108].

*Preclinical Studies.* In vivo and in vitro studies often show that antioxidants can inhibit cancer cell growth and induce apoptosis [109,110]. However, applying these findings to humans is complex because antioxidants’ effects may depend on complex in vivo conditions, such as **a.** the low bioavailability and bio-accessibility in some specific organs, **b.** their concentration and the levels of oxidative stress and hypoxia in the TME, and **c.** their potential pro-oxidant activity. In aerobic conditions, they generate superoxide radicals and dismutate to H_2_O_2_, which reacts with reduced metal ions and superoxide to ROS [111].

### 6.4. Antioxidants in Cancer Treatment

*Chemoprevention*. Antioxidants have been explored as agents to prevent the development of cancer in high-risk populations. For example, retinoids (vitamin A derivatives) are used in the prevention of certain types of skin cancer.

*Adjunct Therapy.* Antioxidants are sometimes used alongside conventional cancer treatments to mitigate side effects and improve overall health. For instance, studies on curcumin and resveratrol have shown promising results in sensitizing cancer cells to chemotherapy [112]. Antioxidants may enhance the efficacy of conventional cancer treatments, such as chemotherapy and radiation, and reduce their side effects by protecting normal cells from oxidative damage [113]. However, there is concern that antioxidants might interfere with the efficacy of chemotherapy and radiation therapy, which rely on generating oxidative stress to kill cancer cells. Ongoing research is evaluating the effects of antioxidants for cancer prevention and as adjuvants in cancer therapy. Table 1 reports active clinical trials testing antioxidants for cancer prevention and treatment.

*Controversies and Considerations.* While antioxidants have potential in the prevention and treatment of age-related cancers, their role is complex and not fully understood. Different antioxidant compounds failed to demonstrate efficacy in clinical practice. Table 2 reports the effects of clinically tested antioxidant supplementation in cancer treatment.

*Pro-oxidant Activity.* At high doses, some antioxidants may have pro-oxidant effects, potentially contributing to oxidative stress and cancer risk.

*Population-Specific Effects.* The impact of antioxidants may vary based on genetic, environmental, and lifestyle factors. What is beneficial for one group may not be for another.

*Dosage and Formulation.* The form and dose of antioxidants are crucial as excessive antioxidant intake might interfere with the oxidative stress required for the effectiveness of certain cancer therapies. Natural sources (e.g., whole fruits and vegetables) are generally preferred over high-dose supplements due to the potential for adverse effects with the latter.

In conclusion, individual variability in response to antioxidants exists, influenced by genetic factors, the type and stage of cancer, and overall health status. Antioxidant compounds hold significant potential in the prevention and treatment of cancer due to their ability to mitigate oxidative stress and modulate various biological processes related to cancer development. While the incorporation of antioxidants from a balanced diet is generally beneficial, further research is needed to fully understand the optimal use of antioxidant supplements in cancer therapy.

## 7. Imaging Techniques to Study Oxidative Damage In Vivo

Studying oxidative damage in vivo requires advanced imaging techniques that can detect and quantify ROS and oxidative damage to biomolecules, such as DNA, proteins, and lipids, providing insights into the related pathological implications in real-time, within living organisms. The main imaging techniques used to study oxidative damage in vivo are the following.

1. **Fluorescence and Bioluminescence Imaging,** which provide sensitive and non-invasive ways to visualize and quantify oxidative stress and related cellular damage, by means of fluorescent probes [128] or bioluminescent reporters [129], in living tissues and organisms [130]:
  - **ROS-sensitive probes** can be used to visualize and quantify ROS in living cells. These probes react with specific ROS to emit fluorescence, which can be detected using fluorescence or confocal microscopy. **Dihydroethidium (DHE)** reacts with O_2_^•−^ to produce fluorescent ethidium, which intercalates into DNA. **2′,7′-Dichlorodihydrofluorescein diacetate (DCFH-DA)** reacts with hydrogen peroxide (H_2_O_2_) to produce a fluorescent product, dichlorofluorescein (DCF). **MitoSOX™** (Thermo Fisher Scientific, Waltham, MA, USA), a mitochondria-targeted version of DHE, specifically detects superoxide production within mitochondria, a key site of oxidative stress.
  - **Lipid Peroxidation Probes, such as BODIPY C11**, are fluorescent dyes that detect lipid peroxidation, a process driven by oxidative stress that damages cell membranes.
  - **Protein and DNA Damage Probes,** such as ***a. Oxidation-Sensitive GFP (roGFP),*** which is a genetically encoded fluorescent protein that changes its fluorescence in response to changes in the redox state of cells, allowing real-time visualization of oxidative stress; and ***b. DNA Damage Sensors*** consisting of fluorescent probes or labeled antibodies that detect 8-oxoguanine, a marker of oxidative DNA damage.
  - **Bioluminescent Reporters** are genetically encoded or chemically synthesized fluorescent proteins that emit light when excited by a specific wavelength of light. These fluorophores can be targeted to specific cells, organelles, or molecules involved in oxidative stress. For example, luciferase-based reporters, where oxidative stress-induced gene expression triggers bioluminescence, allow real-time imaging of oxidative damage [129].
2. **Magnetic Resonance Imaging (MRI)** is a powerful non-invasive imaging technique with excellent spatial resolution and the ability to visualize soft tissues without ionizing radiation. MRI has been adapted to visualize oxidative damage by using redox-sensitive contrast agents or combined with Electron Paramagnetic Resonance (EPR) [131]:
  - **Redox-Sensitive MRI Contrast Agents** that are sensitive to the redox state of tissues can be used to assess oxidative damage. For example, manganese-based contrast agents are oxidized or reduced depending on the oxidative environment, altering their MRI signal.
  - **EPR** is a technique that detects unpaired electrons, such as those in free radicals, making it well-suited for studying oxidative stress [132]. EPR can be combined with MRI to create EPR-MRI, allowing for spatial mapping of free radicals in tissues. Nitroxide-based probes are commonly used in EPR-MRI to detect ROS [133]. These probes are stable free radicals that change their magnetic properties upon reduction by antioxidants or reaction with ROS, providing imaging of oxidative damage in vivo.
3. **Positron Emission Tomography (PET)** is traditionally used to visualize metabolic processes, such as glucose metabolism or blood flow, and can also be applied to study oxidative damage, using specialized radiotracers that target oxidative stress or its related consequences:
  - **PET Imaging with Redox-Sensitive Radiotracers** that are sensitive to redox changes, such as [18F]-labeled dihydroethidine (18F-DHE), and especially [18F]ROStrace, have proven effective for visualizing ROS production and oxidative stress in live models of neuroinflammation [134].
  - **Hypoxia Imaging.** PET imaging with tracers like [18F]FMISO (fluoromisonidazole) is used to study oxidative stress associated with hypoxic conditions (leading to oxidative stress) in ischemic tissues or to identify hypoxic regions of tumors [135], which may be more aggressive and resistant to treatment. PET imaging with ^62^Cu-ATSM, which accumulates in the presence of an over-reductive state, detects oxidative stress in the disease-related brain regions of patients with mitochondrial disease, Parkinson’s disease, and ALS and can be useful for monitoring antioxidant therapies [136].
4. **Optical Imaging (Near-Infrared Fluorescence, NIRF)** takes advantage of the tissue-penetrating properties of near-infrared light and the use of fluorescent probes that can penetrate deep tissues and be activated by ROS or that target specific molecular markers of oxidative stress. ROS-activated probes are non-fluorescent in their native state but become fluorescent upon reacting with ROS, such as H_2_O_2_, O_2_^•−^, or ^•^OH. Examples include **IR-775c**, a near-infrared dye (with absorption and emission typically between 650–900 nm, and good photostability), which becomes fluorescent upon oxidation by ROS, and **Cy7-Based Probes**, a family of cyanine dyes (with absorption typically around 740 nm and emission around 770–790 nm), which are commonly used in NIRF imaging and can be modified to become activated by ROS.
5. **Multiphoton Microscopy** employs two or more photons of low energy (near-infrared light) to excite a fluorophore, which then emits light at a higher energy level. The use of longer wavelength light allows for deeper penetration into tissues and minimizes photodamage, making it ideal for studying processes such as oxidative stress in living cells and tissues [137,138]:
  - **Multiphoton Excitation Microscopy** allows the viewing of oxidative stress-induced changes in cellular structures and can also image ROS by using fluorescent probes, such as dihydroethidium (DHE), which becomes fluorescent when oxidized by superoxide; and MitoSOX™, which is a mitochondria-targeted fluorescent probe that selectively detects superoxide production within mitochondria, a major site of oxidative stress [139].
  - **Multiphoton microscopy** can also detect the intrinsic autofluorescence of naturally occurring fluorophores like NADH/NADPH and flavoproteins (involved in oxidative phosphorylation and used to assess mitochondrial function), whose fluorescence can change in response to oxidative stress, allowing for indirect detection of oxidative damage.
6. **Raman Spectroscopy and Imaging** provide non-invasive, label-free detection of biochemical alterations, making them highly suitable for investigating oxidative modifications in lipids, proteins, and nucleic acids [140] and enabling real-time monitoring of oxidative stress and damage under physiological conditions:
  - **Surface-Enhanced Raman Scattering (SERS)** imaging can detect molecular vibrations that provide detailed information about chemical structures. SERS enhances the Raman signal, enabling the detection of biomolecules involved in oxidative stress, such as lipid peroxidation or protein oxidation, and nucleic acid alterations, providing information on base modifications, backbone damage, and strand breaks.
  - **Label-Free Raman Imaging** can be used to detect oxidative damage to biomolecules without using external probes, thus providing a label-free approach to studying oxidative stress in vivo [141].
7. Super-Resolution Microscopy, which includes STORM (Stochastic Optical Reconstruction Microscopy) and PALM (Photoactivated Localization Microscopy), relies on the precise localization of individual fluorescent molecules. By turning subsets of fluorophores on and off, their exact positions can be calculated, allowing the construction of super-resolution images (at the nanoscale, 20–30 nm) from the aggregated data. STORM and PALM allow for visualizing oxidative damage at the molecular level within cells (cellular organelles, such as mitochondria and endoplasmic reticulum) and are especially useful in studying oxidative damage to DNA or proteins in situ [142].

In conclusion, the study of oxidative damage in vivo relies on a range of imaging techniques, each with its strengths, depending on the type of oxidative damage being studied, the depth of tissue penetration required, and the resolution needed to observe cellular and molecular events in living organisms. These techniques are critical for understanding the role of oxidative stress in diseases such as cancer, neurodegeneration, and cardiovascular disorders, and they also help in evaluating the efficacy of antioxidants and other therapeutic interventions.

## 8. Overgeneration of ROS as Anti-Cancer Therapy

ROS play a dual role in cancer biology. They can promote tumorigenesis at low levels, while at high levels, they cause cell damage and apoptosis [143]. Therefore, ROS overproduction, which can be generated by a variety of tools, including **photodynamic therapy (PDT)** [144] and **sonodynamic therapy (SDT)** [145], is a promising anti-cancer strategy. ROS overproduction for anti-cancer purposes is a subject of active clinical investigation and is especially used in combination therapies with inhibitors of antioxidant pathways (e.g., targeting glutathione or thioredoxin systems) or traditional chemotherapeutics and with radiotherapy (which induce ROS overproduction) to enhance cancer cell killing and achieve synergistic effects. 

Notably, many chemotherapeutic drugs, such as doxorubicin, cisplatin, and paclitaxel, induce ROS production as part of their cytotoxic effects. These drugs either directly generate ROS or disrupt cellular processes that lead to ROS accumulation [146,147].

**Doxorubicin** is an anthracycline chemotherapy drug, widely used to treat a variety of cancers, including breast and lung cancers, lymphomas, leukemias, and sarcomas, due to its ability to intercalate DNA, inhibit topoisomerase II, and generate free radicals [148]. Doxorubicin generates ROS primarily through redox cycling of its quinone moiety. This process involves the enzymatic reduction of the quinone group to a semiquinone radical by NADPH-dependent enzymes, such as NADPH cytochrome P450 reductase or NADH dehydrogenase (Complex I of the electron transport chain). The semiquinone radical is unstable and can rapidly react with O_2_, leading to the formation of O_2_^−^, which can further generate other ROS, such as H_2_O_2_ and OH·, through dismutation and Fenton reactions, respectively. This cycling between the quinone and semiquinone forms can occur repeatedly, amplifying the production of ROS. Doxorubicin accumulates in mitochondria, where it can interact with components of the electron transport chain (ETC), especially Complexes I and III, and disrupt the normal flow of electrons through the ETC, leading to electron leakage and production of O_2_^−^, which in turn generate more ROS.

Alkylating agents, like **cisplatin,** a platinum-based chemotherapeutic used for the treatment of solid tumors [149] such as testicular [150], ovarian [151], bladder [152], lung [153], and head and neck cancers [154] that can bind to and damage mitochondrial DNA (mtDNA), lead to the formation of crosslinks that distort the DNA structure and inhibit transcription of essential components of the ETC. Cisplatin can also directly impair the function of the ETC, leading to electron leakage from the mitochondrial inner membrane and consequent overproduction of ROS, particularly O_2_^−^. Highly reactive ROS can cause oxidative damage to lipids, proteins, and mitochondrial membranes, further exacerbating mitochondrial dysfunction.

**Paclitaxel** (Taxol) is a widely used chemotherapeutic agent primarily for the treatment of solid tumors, including breast [155], ovarian [156], and lung [157] cancers, which exerts its antitumor effects by stabilizing microtubules, preventing their depolymerization, and thereby disrupting cell division. Beyond its effects on the cytoskeleton, paclitaxel induces ROS overproduction [158] through several mechanisms that contribute to both its therapeutic efficacy and its side effects. Paclitaxel induces mitochondrial dysfunction by disrupting the ETC, leading to the leakage of electrons from the ETC, which can prematurely react with molecular oxygen to form O_2_^•−^. These superoxides can be further converted into other ROS like H_2_O_2_ and ^•^OH radicals. Furthermore, paclitaxel causes changes in the mitochondrial membrane potential (ΔΨm), leading to mitochondrial depolarization and dysfunction, further increasing ROS production. Additional mechanisms of paclitaxel-induced ROS overproduction include endoplasmic reticulum stress, due to the accumulation of misfolded proteins within the endoplasmic reticulum, which triggers the unfolded protein response (UPR) and activates pathways that increase the production of ROS. Endoplasmic reticulum stress also leads to the release of calcium ions (Ca^2+^) from the endoplasmic reticulum into the cytosol, resulting in abnormal calcium signaling, which can exacerbate mitochondrial dysfunction, amplifying ROS production.

ROS overproduction is central to the mechanism of action of **PDT as an anti-cancer treatment** [144,159]. PDT is a non-invasive therapeutic approach that uses **1. a light-sensitive compound** (non-toxic until activated by light) known as photosensitizers, which is administered to the patient and accumulates preferentially in cancer cells; **2. light exposure** (specific wavelength of light, usually in the visible or near-infrared spectrum) required to activate the photosensitizer; and **3. molecular oxygen** present in the tissue, to generate ROS, leading to the selective destruction of cancer cells. The most significant ROS produced in PDT is ^1^O_2_, O_2_^−^, ^•^OH, and hydrogen peroxide (H_2_O_2_), which can also be generated depending on the environment and the type of photosensitizer used.

**The advantages of PDT** consist of **a. selective targeting** since the production of ROS is localized to the site of light exposure, allowing for precise targeting of the tumor while minimizing damage to surrounding healthy tissues, **b. low risks of systemic toxicity** since the photosensitizer remains relatively inert until activated by light, and **c. efficiency in overcoming tumor resistance to conventional treatments** since the types of cellular damage caused by ROS in PDT are different from those caused by traditional chemotherapy or radiotherapy, which makes PDT a powerful tool in cancer therapy, especially for surface or accessible tumors. PDT is mainly used for localized tumors and is particularly effective for certain types of cancers and non-cancerous conditions, such as skin cancers (basal cell carcinoma and squamous cell carcinoma in situ), pre-cancerous skin conditions (actinic keratosis), early-stage esophageal cancer, early-stage bladder cancer, and cervical intraepithelial neoplasia (CIN) [160]. However, challenges such as oxygen dependence and light penetration still need to be addressed to fully realize the potential of PDT in broader clinical applications.

**SDT is an emerging cancer treatment** that combines ultrasound with a sonosensitizer to produce ROS and induce selective tumor cell death [145]. As in the case of PDT, SDT relies on ROS as the primary agents of cytotoxicity, but it uses ultrasound, typically in the low-frequency range (20 kHz to 3 MHz), instead of light to activate the sensitizer, a compound that is sensitive to ultrasound, such as Hematoporphyrin Derivatives (HPDs) or Protoporphyrin IX (PpIX) or doxorubicin, which is administered to the patient. The ultrasound waves activate the sonosensitizer, leading to the generation of ROS such as singlet oxygen and free radicals, and cause damage to cellular components like DNA, proteins, and lipids, ultimately leading to cancer cell death. The selective accumulation of sonosensitizers in tumor tissues, combined with the localized application of ultrasound, allows for targeted cancer treatment with minimal damage to surrounding healthy tissues.

The advantage of SDT lies in its ability to treat deeply located tumors. On the other hand, photodynamic therapy uses visible light, which attenuates rapidly in tissues, has limited penetration, can be employed only superficially or intra-operatively, and is not as effective as therapy in the deeper regions of the tumor [145]. SDT recently emerged as a promising noninvasive alternative to chemotherapy and radiation therapy in the treatment of aggressive gliomas. Studies have suggested that coupling sonosensitizers such as 5-aminolevulinic acid, hematoporphyrin monomethyl ether, and sinoporphyrin sodium with focused ultrasound induces robust cytotoxic activity in tumor cells, in vitro and in vivo. These effects are likely mediated by the oxidative stress induced by reactive oxygen species production, apoptotic signaling cascades, and intracellular calcium overload [161].

SDT is currently under investigation in preclinical and early clinical studies. Researchers are focusing on optimizing sonosensitizer formulations, improving ultrasound delivery methods, and understanding the mechanisms of ROS generation and tumor destruction. Some sonosensitizers, such as porphyrins and nanoparticles, are being tested for their efficacy in specific cancer types, including hepatocellular carcinoma and pancreatic adenocarcinoma, which are often resistant to conventional therapies [162].

## 9. Understanding the Mechanisms Underlying the Oxidative Stress–Aging–Carcinogenesis Relationships to Fight Age-Related Tumors

Geroscience is only beginning to inform oncology of the relationship between oxidative stress, aging, and cancer. It has been established that oxidative stress, in the form of excess ROS or RNS, promotes both aging and cancer development by causing structural damage in intracellular proteins and lipids and by mutating nuclear or mitochondrial DNA. Prostate, breast, lung, and colorectal cancer incidence increases with age. However, there is epidemiological evidence that cancer incidence and aggressiveness decrease after the age of 80 [163,164], contradicting the aging–cancer paradigm and raising questions that need to be urgently addressed given the worldwide aging population and the demand for healthy aging.

### 9.1. Cancers Associated with Oxidative Stress and Aging

*Lung Cancer.* The lung is constantly exposed to higher oxygen pressures compared to other tissues [165], therefore making it a major target for air pollutants, many of which are electrophilic molecules that can induce ROS production [166,167]. Cigarette smoke is a major source of oxidative stress [168] and can significantly increase the production of ROS in the lung parenchyma, which can, in turn, induce IL-8 release and SNAIL1 expression in cancer cells, leading to the epithelial–mesenchymal transition, which promotes tumor progression [169].

The accumulation of harmful peroxides and lipid peroxidation products in the lung parenchyma is also promoted by the impairment of antioxidant defense mechanisms [170]. In squamous cell lung carcinoma and adenocarcinoma, the activity of glutathione peroxidase and catalase is significantly decreased, compared to normal lung tissue, and this is accompanied by a significant increase in reactive aldehydes [170], which can induce high levels of oxidative stress.

The presence of massive oxidative damage in lung cancer patients is also demonstrated by the elevated levels of oxidative stress markers, such as protein carbonylation and malondialdehyde (MDA)-protein adducts, in the bronchial epithelium and peripheral blood [171].

*Breast Cancer.* Oxidative stress has been implicated in breast carcinogenesis in relation to age-related hormonal changes [172] and environmental factors [173]. In particular, the relationship between estrogen metabolism and oxidative stress in breast cancer involves various mechanisms and pathways.

Estrogens can increase mitochondrial ROS production by down-regulating uncoupling proteins (UCPs), leading to oxidative stress in estrogen receptor (ER)-positive breast cancer cells [174,175]. This oxidative stress is implicated in the onset and progression of breast cancer through the formation of DNA adducts and subsequent mutations [176,177,178]. In addition, chronic oxidative stress can lead to epigenetic changes, such as the inactivation of the ERα gene, contributing to the development of more aggressive, estrogen-independent breast cancer subtypes [179].

*Colorectal Cancer.* Diet, chronic inflammation, and gut microbiota dysbiosis [180] can contribute to oxidative stress in the colon. ROS-induced DNA damage in colonic epithelial cells is a critical step in colorectal cancer development [181].

Epidemiological and experimental studies have proven that a high-fat diet is an important risk factor for colon cancer [182], particularly due to the interactions between dietary fats, gut microbiota, and inflammatory processes. High-fat diets can promote obesity, resulting in insulin resistance and inflammation and the development of oxidative stress, increased cell proliferation, and suppression of apoptosis [182].

Increased levels of oxidative DNA damage markers, such as 8-hydroxy-2′-deoxyguanosine (8-OHdG), are observed in colon cancer tissues, compared to normal colonic mucosa [183]. In addition, the altered function of enzymes involved in the repair of oxidative DNA damage, e.g., 8-oxoguanine DNA N-glycosylase 1 (hOGG1) and mutY DNA glycosylase (MUTYH), results in the accumulation of DNA mutations and contributes to the adenoma-adenocarcinoma transition [184].

The gut microbiota can also be involved in the development of oxidative stress since the microbial expression of ROS-scavenging enzymes is compromised in cancer. This evidence suggests that the gut microbiota, under normal conditions, may protect the colonic mucosa from oxidative stress and that the impairment of this activity may contribute to the onset and progression of colon cancer [185].

*Prostate Cancer.* Oxidative stress plays a role in the initiation and progression of prostate cancer. Age-related increases in ROS production and decreased antioxidant defenses are contributing factors [186].

The serum levels of protein oxidation and lipid peroxidation products increase in prostate cancer patients with a Gleason score >7 compared to patients with a Gleason score <7, and the presence of perineural invasion significantly is associated with an increase in the concentration of protein oxidation products in the urine [187]. These results suggest a significant role for oxidative damage in prostate cancer onset and progression [187]. Furthermore, oxidative stress can induce, in prostate cancer cells, the expression of genes such as Lanthionine synthase C-like protein 1 (LanCL1) [188], which promotes tumor growth and progression, and protects cells from damage caused by oxidative stress resulting in resistance to therapies [188].

*Liver Cancer.* Conditions such as hepatitis, alcohol abuse, and non-alcoholic fatty liver disease can lead to chronic oxidative stress in the liver. This persistent oxidative damage promotes hepatocellular carcinoma [189,190].

Due to their high metabolic activity, hepatocytes are characterized by a high number of mitochondria that serve as sources of ROS. ROS overproduction is a common occurrence in chronic liver diseases and leads to DNA damage and inflammatory infiltration, which collectively exacerbate liver damage and promote malignant transformation [191,192]. Analyses of serum markers and histological findings indicate that oxidative stress increases the likelihood of developing hepatocarcinoma [193]. Oxidative damage can also promote the progression of hepatocellular carcinoma since it can induce the degradation of E-cadherin, mediated by ring finger protein 25 (RNF25), which is associated with tumor metastases [194].

*Skin Cancer.* As the greatest defense organ of the body, the skin is continuously exposed to endogenous and external factors that can induce the production of ROS [195]. Ultraviolet (UV) radiation from the sun is the primary source of ROS in skin cells. UV-induced oxidative stress is a major cause of skin cancers, including melanoma [196] and non-melanoma skin cancers [197].

Two main mechanisms underlie oxidative stress-induced skin cancers: **1.** the direct degradation of proteins, DNA, and lipids by ROS, and **2.** the alteration of cell signaling pathways, such as MAPK and JAK/STAT [195]. ROS can be involved in all stages of carcinogenesis, including initiation, promotion, and progression [198]. Oxidative stress parameters (superoxide anion radical levels and manganese superoxide dismutase and catalase activity) are significantly higher in melanoma patients than in healthy controls and increase with the disease progression, achieving the maximum in stage IV [199]. Patients with nonmelanoma skin cancer have high blood levels of all oxidative stress biomarkers compared to healthy subjects [200].

It has been recently demonstrated that *Staphylococcus aureus* strains isolated from biopsies of cutaneous squamous cell carcinoma can secrete molecules, which can have cancer-promoting effects as they are able to induce the ROS production and DNA damage in keratinocytes [201].

### 9.2. Strategies to Mitigate Oxidative Stress or Improve Antioxidant Defenses

Current strategies to combat oxidative stress focus on reducing the production of ROS, enhancing antioxidant defenses, and repairing or removing damaged molecules. These strategies span from lifestyle modifications to pharmaceutical interventions and emerging biotechnologies.

*Lifestyle Modifications.* Consuming a diet rich in natural antioxidants helps neutralize ROS. Foods rich in vitamins C and E, carotenoids (e.g., beta-carotene, lycopene), polyphenols (e.g., flavonoids in fruits and vegetables), and minerals like selenium and zinc play a key role in reducing oxidative stress. A diet rich in fruits, vegetables, and whole grains can enhance the body’s antioxidant defenses [202]. Supplements or compounds that enhance levels of endogenous antioxidants, such as ***a. N-acetylcysteine (NAC),*** which boosts glutathione production and can be administered orally (typically given over 72 h) or intravenously (given over 20–24 h), is particularly useful in treating acetaminophen (paracetamol) overdose and hepatotoxicity. Its mucolytic and antioxidant properties make it useful in managing respiratory illness, liver diseases [203], and psychiatric disorders [204]; ***b. Coenzyme Q10,*** also known as ubiquinone, supports mitochondrial antioxidant defenses and is often used as an adjunctive therapy in patients with heart failure, hypertension, ischemic heart disease, and neurological disorders for its potential neuroprotective effects [205].

Caloric restriction has been shown to reduce oxidative stress and extend lifespan in a variety of organisms [206,207], decreasing the overall metabolic rate and improving mitochondrial efficiency, meaning that less ROS is produced per unit of energy generated. Regular physical activity and avoiding smoking [166] and excessive alcohol consumption [208] can reduce oxidative stress. Limiting exposure to environmental pollutants (e.g., air pollution, heavy metals) and UV radiation can reduce oxidative stress by minimizing external sources of ROS.

*Pharmacological Interventions.* Drugs that mimic natural antioxidants or neutralize ROS directly are being developed. For example, **edaravone**, also known by its chemical name MCI-186, is a free-radical scavenger used as a neuroprotective agent to treat oxidative stress-related conditions like acute ischemic stroke and ALS [209,210]. Edaravone is administered intravenously, which ensures high bioavailability, and it is rapidly metabolized in the liver and excreted through the kidneys, primarily as glucuronide and sulfate conjugates. For the treatment of ALS, edaravone is administered as an i.v. infusion at a dose of 60 mg per day for 10–14 days, followed by a break, and then a continuation with a similar on–off cycle. In the treatment of acute ischemic stroke, edaravone is given at 30 mg twice daily for 14 days, proving more effective when given within 24 h of stroke onset. Although edaravone is primarily an antioxidant, excessive dosing or specific biochemical conditions (e.g., metal ion interactions since edaravone can interact with metal ions such as iron and copper, potentially catalyzing redox reactions and more free radical production) could result in pro-oxidant effects. Careful monitoring and personalized dosing strategies can help mitigate the risks associated with promoting oxidation.

Due to its antioxidant and anti-inflammatory properties, edaravone has also been investigated in preclinical models for its potential activities in treating cancer [211].

Additional drugs targeting oxidative stress pathways are being investigated for cancer prevention and treatment, including inhibitors of ROS-producing enzymes, such as NOX Inhibitors [212], Cyclooxygenase (COX) Inhibitors [213], Lipoxygenase (LOX) Inhibitors [214], and agents that boost endogenous antioxidant defenses [215]. Since chronic inflammation is often associated with increased ROS production, anti-inflammatory drugs such as nonsteroidal anti-inflammatory drugs, NSAIDs, and corticosteroids can indirectly reduce oxidative stress by curbing inflammation.

*Gene and Molecular Therapy.* These biotechnological approaches aim to enhance the expression of endogenous antioxidant enzymes like SOD, catalase, and GPx to reduce oxidative stress. Gene editing tools like CRISPR/Cas9 and RNA-based therapies, e.g., Small Interfering RNA (siRNA) and antisense oligonucleotides (ASOs) are being explored to specifically target genes involved in oxidative stress pathways, potentially offering new therapeutic approaches for diseases associated with oxidative damage.

- **The CRISPR/Cas9 system** allows for precise editing of genes that contribute to oxidative stress, such as Kelch-like ECH-associated protein 1 (Keap1), which regulates the activity of Nrf2 [216] and other redox regulators. CRISPR has been used to knock out mutant forms of proteins that lead to oxidative damage, offering a potential cure for neurodegenerative diseases (Alzheimer’s, Parkinson’s, and ALS), cardiovascular disorders, and cancer.
   In neurodegenerative diseases, CRISPR has been used to selectively knock out the mutant form of the huntingtin (HTT) gene, reducing the toxic protein burden and mitigating oxidative stress in neuronal cells [217]. Mutations in the SOD1 gene are implicated in familial ALS. CRISPR/Cas9 has been used to knock out the mutant SOD1 gene in cellular and animal models of ALS, reducing oxidative stress and extending survival in preclinical studies [218]. Mutations in the PARK7 (DJ-1) gene are associated with oxidative damage in Parkinson’s Disease. CRISPR/Cas9 has been employed to knock out these mutant genes or restore normal gene function, reducing oxidative stress and improving cellular survival in experimental models [219].
   In cardiovascular diseases, including atherosclerosis and ischemia-reperfusion injury, which are characterized by oxidative stress-induced damage to the vascular endothelium and heart tissue, CRISPR/Cas9 has been used to target genes that contribute to ROS production or impair antioxidant defenses. CRISPR/Cas9-mediated knockout of the PCSK9 gene, which plays a role in cholesterol metabolism and oxidative stress in vascular cells, has been explored as a therapeutic strategy to reduce oxidative damage in atherosclerosis, with promising results in animal models [220].
   In the oncology field, CRISPR/Cas9 is being used to target genes that exacerbate oxidative damage in cancer cells, either by knocking out pro-oxidant genes or modulating antioxidant defenses. CRISPR/Cas9-mediated knockout mutant forms of the TP53 gene restore normal redox balance and reduce oxidative damage in cancer cells [221]. Glutathione (GSH) is a major antioxidant in cells. CRISPR/Cas9 has been used to knock out genes like the Glutamate–cysteine ligase catalytic subunit, GCLC (a key enzyme in glutathione synthesis), in Acute myeloid leukemia (AML) and solid tumors [222] to increase their sensitivity to oxidative damage, making them more susceptible to chemotherapy and radiotherapy.
- **siRNA** or **ASOs** can silence genes involved in ROS production or modulate antioxidant pathways. NOX enzymes are key contributors to ROS production. siRNA or ASOs targeting NOX subunits can reduce ROS levels in various cell types. siRNA-targeting NOX2 has shown promise in reducing oxidative stress in models of cardiovascular disease and neuroinflammation [223,224,225].
   The mechanistic target of rapamycin (mTOR) pathway regulates various cellular processes, including oxidative stress. siRNA-targeting components of the mTOR pathway, such as mTOR itself or downstream effectors, can modulate ROS production in cancer and metabolic disorders [226,227]. siRNA or ASOs targeting negative regulators of SOD (e.g., SOD1 mutations in ALS) aim to enhance SOD activity indirectly by silencing genes that inhibit its function [228,229].
   GPx enzymes reduce hydrogen peroxide to water. siRNA targeting genes involved in the regulation or production of GPx (e.g., GPx4) [230] can enhance the cellular antioxidant capacity in models of cancer and neurodegenerative diseases [231,232].
   Nrf2 is a key transcription factor that regulates antioxidant gene expression. siRNA can target negative regulators of Nrf2 (e.g., Keap1) to increase the activity of Nrf2 and enhance antioxidant responses. In diseases where Nrf2 is dysfunctional, such as autoimmune (Multiple Sclerosis), chronic inflammatory diseases (Rheumatoid Arthritis), and neurodegenerative disorders [233], siRNA can help restore its normal function.
   While gene and molecular therapies hold significant promise in developing therapies for oxidative stress, further studies are needed to overcome challenges related to delivery, immune responses, off-target effects, and safety concerns such as the risk of insertional mutagenesis or unintended gene activation.

*Microbiome Modulation.* The gut microbiome plays a pivotal role in modulating oxidative stress through various mechanisms, including the production of antioxidant compounds, regulation of inflammation, and maintenance of mitochondrial health. Dysbiosis can lead to the overproduction of ROS by pathogenic bacteria and the immune system, increasing oxidative stress [234]. A healthy and balanced gut microbiome can help reduce systemic oxidative stress and protect against a range of chronic diseases. Certain probiotic strains, such as Lactobacillus rhamnosus and Bifidobacterium breve, have been shown to reduce oxidative stress by enhancing antioxidant defenses and reducing pro-inflammatory cytokines [235,236]. Dietary fibers that act as prebiotics (e.g., inulin, fructooligosaccharides) promote the growth of beneficial bacteria that produce SCFAs, thereby enhancing antioxidant defenses and reducing oxidative stress [237,238,239].

Overall, oxidative stress is a significant factor in the development and progression of age-related cancers, and its modulation could improve anti-tumor treatments [240]. While antioxidant therapies and lifestyle modifications hold promise, ongoing research is crucial to fully elucidate the complex relationship between oxidative stress, aging, and cancer to reduce the incidence and impact of age-related malignancies.

## Figures and Tables

**Table 1:** Active clinical trials testing antioxidants for cancer prevention and treatment.

NCT Number	Study Title	Conditions	Phases
NCT02891538	Chemopreventive Effects of Epigallocatechin Gallate (EGCG) in Colorectal Cancer (CRC) Patients	Colon Cancer	EARLY PHASE1
NCT05932511	Topical Ascorbic Acid for Treatment of Squamous Cell Skin Cancer	Squamous Cell Cancer; Squamous Cell Carcinoma; Skin Cancer; Non-melanoma Skin Cancer	EARLY PHASE1
NCT05985278	Clinical Application of Lutetium [177Lu]-Catalase in Tumor Radionuclide Therapy	Advanced Malignant Neoplasm	EARLY PHASE1
NCT01752491	A Phase I Trial of High-Dose Ascorbate in Glioblastoma Multiforme	Glioblastoma; GBM; Glioblastoma Multiforme	PHASE1
NCT01852890	Gemcitabine, Ascorbate, Radiation Therapy for Pancreatic Cancer	Pancreatic Neoplasms	PHASE1
NCT03278925	Defined Green Tea Catechin Extract in Preventing Liver Cancer in Participants With Cirrhosis	Cirrhosis	PHASE1
NCT03602235	High Dose Ascorbic Acid for Plasma Cell Disorders	Multiple Myeloma	PHASE1
NCT04900792	A Safety Study of Pharmacologic Ascorbate and Ferumoxytol in Addition to Standard of Care Chemoradiation in Glioblastoma	Glioblastoma; Glioblastoma Multiforme	PHASE1
NCT04952129	Optimal Selenium for Bowel Polyps (OSCAR)	Colorectal Adenoma	PHASE1
NCT05081479	A Study of N-Acetylcysteine (N-AC)in People Receiving CAR T cell Therapy for Lymphoma	Lymphoma	PHASE1
NCT04046094	Intravenous (IV) Vitamin C With Chemotherapy for Cisplatin Ineligible Bladder Cancer Patients	Bladder Cancer	PHASE1 PHASE2
NCT05123365	An Optimal Dose Finding Study of N-Acetylcysteine in Patients With Myeloproliferative Neoplasms	Myeloproliferative Neoplasm; MPN; Essential Thrombocythemia; Polycythemia Vera; Myelofibrosis	PHASE1 PHASE2
NCT05363631	Seleno-L Methionine (SLM)-Axitinib-Pembrolizumab	Clear Cell Renal Cell Carcinoma; Clear Cell Renal Cell Carcinoma Metastatic	PHASE1 PHASE2
NCT05721872	Efficacy, Tolerability and Safety of Intravenous D-VC With ATO in Patients With Advanced/Metastatic Colorectal Cancer	Colorectal Cancer; Metastatic Colorectal Cancer	PHASE1 PHASE2
NCT01871454	Safety of Pentoxifylline and Vitamin E With Stereotactic Ablative Radiotherapy (SABR) in Non-small Cell Lung Cancers	Non-small Cell Lung Cancers	PHASE2
NCT02344355	A Phase 2 Trial of High-Dose Ascorbate in Glioblastoma Multiforme	Glioblastoma Multiforme	PHASE2
NCT02705300	Side Effects to FOLFOXIRI + Tocotrienol/Placebo as First Line Treatment of Metastatic Colorectal Cancer	Colorectal Cancer	PHASE2
NCT02905578	A Phase 2 Trial of High-dose Ascorbate for Pancreatic Cancer (PACMAN 2.1)	Pancreatic Neoplasms; Cancer of Pancreas; Cancer of the Pancreas; Neoplasms, Pancreatic; Pancreas Cancer; Pancreas Neoplasms; Adenocarcinoma	PHASE2
NCT02905591	A Phase 2 Study Adding Ascorbate to Chemotherapy and Radiation Therapy for NSCLC	Carcinoma, Non-Small-Cell Lung; Non-Small Cell Lung Cancer; Nonsmall Cell Lung Cancer; Non-Small-Cell Lung Carcinoma; NSCLC	PHASE2
NCT03418038	Ascorbic Acid and Chemotherapy for the Treatment of Relapsed or Refractory Lymphoma, CCUS, and Chronic Myelomonocytic Leukemia	Clonal Cytopenia of Undetermined Significance; High Grade B-Cell Lymphoma With MYC and BCL2 or BCL6 Rearrangements; Recurrent Diffuse Large B-Cell Lymphoma; Recurrent Hodgkin Lymphoma; Recurrent Lymphoma; Refractory Diffuse Large B-Cell Lymphoma; Refractory Lymphoma; Chronic Myelomonocytic Leukemia	PHASE2
NCT03476330	Quercetin Chemoprevention for Squamous Cell Carcinoma in Patients With Fanconi Anemia	Fanconi Anemia; Squamous Cell Carcinoma	PHASE2
NCT04033107	High Dose Vitamin C Combined With Metformin in the Treatment of Malignant Tumors	Hepatocellular Cancer; Pancreatic Cancer; Gastric Cancer; Colorectal Cancer	PHASE2
NCT04175470	Bevacizumab and Tocotrienol in Recurrent Ovarian Cancer	Ovarian Cancer Recurrent	PHASE2
NCT04245865	Tocotrienol and Bevacizumab in Metastatic Colorectal Cancer	Colorectal Cancer Metastatic	PHASE2
NCT04801511	Preoperative IMRT With Concurrent High-dose Vitamin C and mFOLFOX6 in Locally Advanced Rectal Cancer	Rectal Cancer	PHASE2
NCT05724329	Neoadjuvant Tislelizumab in Combination With Dasatinib and Quercetin in Resectable HNSCC (COIS-01)	Head and Neck Squamous Cell Carcinomas	PHASE2
NCT06087237	The Efficacy of Using Pentoxifylline in Patients Undergoing Breast Cancer Surgery	Post-Surgical Complication; Breast Cancer Surgery	PHASE2
NCT06176339	Assessing the Clinical Utility of Adding Pentoxifylline to Neoadjuvant Chemotherapy Protocols in Breast Cancer Patients”	Breast Cancer	PHASE2
NCT06355037	Dasatinib Combined With Quercetin to Reverse Chemo Resistance in Triple Negative Breast Cancer	Triple-negative Breast Cancer	PHASE2
NCT06398405	A Phase II Clinical Study of Epigallocatechin-3-gallate in Patients With Esophageal Squamous Cancer	Esophageal Cancer; Dysphagia, Esophageal; Epigallocatechin Gallate	PHASE2
NCT06115408	Evaluation of Using Dienogest and N-Acetyl Cysteine on the Volume of Uterine Leiomyoma	Uterine Leiomyoma	PHASE2 PHASE3
NCT05364008	FRIEND: Fibroids and Unexplained Infertility Treatment With Epigallocatechin Gallate; A Natural CompounD in Green Tea	Uterine Leiomyoma	PHASE3
NCT05448365	Vitamin D, Epigallocatechin Gallate, D-chiro-inositol and Vitamin B6 in Uterine Fibroid	Uterine Fibroids; D-chiro-inositol; Inositol; Epigallocatechin Gallate; Vitamin D; Vitamin B6	PHASE3
NCT05502900	Adjuvant Melatonin for Uveal Melanoma	Uveal Melanoma; Uveal Melanoma, Posterior, Medium/Large Size; Eye Cancer, Intraocular Melanoma	PHASE3
NCT05631041	Effect of Silymarin in Metastatic Colorectal Cancer Patients	Metastatic Colorectal Cancer	PHASE3

**Table 2:** Effects of antioxidant supplementation in cancer treatment.

Antioxidants	Conditions	Dosage and Additional Substances	Results
*Phytosterols*	Breast cancer	920 g/week of extra virgin olive oil	62% decrease in the risk of malignant breast cancer [114]
		30 g/day of mixed nuts	Non-significant decrease in the risk of breast cancer [114]
*Beta-carotene*	Lung cancer	20 mg/day	Inverse association between dietary intake and the risk of lung cancer; higher mortality among recipients of beta carotene [115]
		20 mg + 50 mg/day of alpha-tocopherol	
	Breast cancer	Daily intake of 5 vegetable servings, 454 g of vegetable juice or vegetable equivalents, 3 fruit servings, 30 g fiber, and 15–20% energy intake from fat	21% decrease in the risk of malignant breast cancer [116]
	Cervical intraepithelial neoplasia (CIN)	30 mg/day	Non-significant effect [117]
		30 mg/day	Response rate of 70% at 6 months and 43% at 12 months [118]
	Basal-cell carcinoma (BCC) Squamous-cell carcinoma (SCC)	30 mg/day + and a sun protection factor 15+ sunscreen	Slightly lower incidence for BCC (not significant); Slightly higher incidence of squamous-cell carcinoma (SCC) (not significant) [119]
		15 mg + 0.3 g vitamin C	
		30 mg/day	
	Lung cancer	30 mg + 25,000 IU retinil palmitate	Lack of chemopreventive benefit and increase of lung cancer incidence and mortality [120,121,122]
	Non-melanoma (NMSC) skin cancer	50 mg/day	No effect on the incidence of NMSC [123]
		50 mg on alternate days	No effect on the incidence of the first NMSC, including BCC and SCC, prevention [124]
*Resveratrol*	Multiple myeloma (MM)	5 g/day	Minimal efficacy in patients with relapsed/refractory MM; Poorly tolerated side effects [125]
	Prostate cancer	Curcumin 0.1 g/day; resveratrol 30 mg/day; catechins 0.1 g/day; fresh broccoli sprouts equivalent to 2 g/day	Non-significant increase in the slope of PSA [126]
*Muscadine Grape Skin Extract (MPX)*	Prostate cancer	4 g/day of MPX, 0.5 g/day of MPX (1.2 mg of ellagic acid, 9.2 μg of quercetin, and 4.4 μg of trans-resveratrol)	No significant difference in PSA Doubling Time [127]
		1.2 mg of ellagic acid, 9.2 μg of quercetin, and 4.4 μg of trans-resveratrol	

## Data Availability

No new data were created or analyzed in this study. Data sharing is not applicable to this article.
